# Positive Cofactor 4 (PC4) is critical for DNA repair pathway re-routing in DT40 cells

**DOI:** 10.1038/srep28890

**Published:** 2016-07-04

**Authors:** Randolph B. Caldwell, Herbert Braselmann, Ulrike Schoetz, Steffen Heuer, Harry Scherthan, Horst Zitzelsberger

**Affiliations:** 1 Helmholtz Zentrum München - German Research Center for Environmental Health (GmbH). Department of Radiation Sciences - Research Unit Radiation Cytogenetics, Ingolstädter Landstraße 1, 85764 Neuherberg, Germany; 2 Clinical Cooperation Group ‘Personalized Radiotherapy of Head and Neck Cancer’, Helmholtz Zentrum München, Ingolstaedter Landstr 1, 85764, Neuherberg, Germany; 3 Department of Radiotherapy and Radiation Oncology, Ludwig-Maximilians-University Munich, Marchioninistr 15, 81377, Munich, Germany; 4 Bundeswehr Institute of Radiobiology affiliated to the University of Ulm. Neuherbergstr. 11, 80937 Muenchen, Germany

## Abstract

PC4 is an abundant single-strand DNA binding protein that has been implicated in transcription and DNA repair. Here, we show that PC4 is involved in the cellular DNA damage response. To elucidate the role, we used the DT40 chicken B cell model, which produces clustered DNA lesions at Ig loci via the action of activation-induced deaminase. Our results help resolve key aspects of immunoglobulin diversification and suggest an essential role of PC4 in repair pathway choice. We show that PC4 ablation in gene conversion (GC)-active cells significantly disrupts GC but has little to no effect on targeted homologous recombination. In agreement, the global double-strand break repair response, as measured by γH2AX foci analysis, is unperturbed 16 hours post irradiation. In cells with the pseudo-genes removed (GC inactive), PC4 ablation reduced the overall mutation rate while simultaneously increasing the transversion mutation ratio. By tagging the N-terminus of PC4, gene conversion and somatic hypermutation are all but abolished even when native non-tagged PC4 is present, indicating a dominant negative effect. Our data point to a very early and deterministic role for PC4 in DNA repair pathway re-routing.

Ionizing radiation induces clustered DNA damage, including abasic sites, and single- and double-strand breaks (SSB and DSB). This type of locally clustered lesions is also a hallmark of somatic diversification in the adaptive immune response. Immunoglobulin (Ig) and T cell receptor (TCR) diversification require a delicate coordination of targeted DNA damage and repair/misrepair. Faithful DNA repair is crucial to maintain genome integrity and chromosomal stability, but is incompatible with the mechanism of normal Ig and TCR gene diversification, where mutations leading to diversity are highly desirable. It is generally accepted that activation-induced deaminase (AID) is required to initiate the process of Ig gene diversification via the creation of lesions^1^. This is accomplished by the deamination of cytosines in exposed ssDNA at Ig loci. However, locus-specific targeting and resulting repair/misrepair is poorly understood, and deamination of cytosine residues alone cannot explain the spectrum of mutations observed^2^.

The primary repair pathway for a cytosine deamination event is the normally faithful short patch Base Excision Repair (BER) pathway, which converts the uracil via uracil-DNA glycosylase (UNG) to an abasic site that is then excised and replaced with the correct nucleotide^3^. This repair pathway functions within the Ig loci, as has been demonstrated by the suppression or disruption of UNG, leading to altered transition:transversion (trs:trv) mutation ratios and reduced gene conversion (GC)^4^,^5^. This suggests that under normal circumstances, faithful BER repair is predominant at Ig loci unless re-routed into an alternative mechanism such as translesion synthesis (TLS) or the mismatch repair (MMR) pathway using an error-prone polymerase^1^. In contrast to what might be expected, the increased C:G → U:G → T:A transition mutations suggest that MMR does not play a major role in the resolution of the U:G mismatched pairs. Furthermore, it has been reported that MMR deficiency in DT40 cells has little to no effect on normal Ig diversification activity outcomes^6^. Nevertheless, while TLS or mispaired base incorporation may account for point mutations, an inordinate amount of localized lesions would have to occur to achieve the spectrum of the mutational diversity seen, considering the deamination rate being estimated at only 70–200 events/cell/day^7^ and the overall creation of abasic sites is estimated at 70,000–100,000 per cell/day^8^ in humans. In animals such as rabbits, cows and chickens, Ig diversity is driven by the same AID deamination event, but followed by GC using pseudo-genes (ψVL1–ψVL25 in DT40) as templates to obtain a critical mass of repair-induced variation^9^. While GC is not known to be involved in human immunoglobulin diversification, it does play a role in human genome evolution and disease where, e.g., DNA repair proteins such as BRCA1 and BRCA2 interact with the homology-searching/strand-pairing protein RAD51, and the MRE11A–RAD50–NBN (MRN) complex^10^,^11^. Homology-directed repair (HDR) such as GC is thought to require double-strand breaks (DSBs) and Rad51 loading on resected ssDNA, thereby allowing strand invasion and homology searching^12^. DSBs can occur if just a few lesions in close proximity on opposite strands are repaired by long-patch BER or Nucleotide Excision Repair (NER); the resected DNA created by these pathways can lead to repair-induced DSBs^13^,^14^. NER, with its sub-pathways of transcription-coupled repair (TCR) and global genome repair (GGR), is thought to have little or no effect on somatic hypermutation and limited effect on class switch recombination^15^,^16^. However, the NER-associated 3′ endonuclease protein XPG is suspected of recruiting PC4 to bind ssDNA, resulting in displacement of XPG^17^. This could represent a mechanism to switch repair pathways in the presence of concurrent opposite strand damage, e.g. lesion clusters, and/or other factors. It is worth considering that employing various activity-switching mechanisms, perhaps useful in facilitating redundant back-up repair systems, may play a role in interrupting normal repair. This kind of mechanism is speculated to be driven by Poly (ADP-Ribose) Polymerase 1 (PARP1), which plays a role in single-strand break (SSB) repair, but also interferes with Ku-mediated non-homologous end joining (NHEJ) repair of DSBs by interacting directly with Ku70 in a non-DNA dependent manner^18^. In *PARP-1*^−/−^ DT40 cells, GC is reduced, but is restored in the PARP1-Ku70 double knockout^18^. Adding to the complexity of possible switching mechanisms are the recently reported findings that a ∆MRN (truncated NBN) complex defective in homologous recombination repair reduces GC^19^. This defect could be rescued by a 3′-to-5′ single strand exonuclease (*Escherichia coli* sbcB), but not by overexpression of the eukaryotic EXO1^19^. Taken together, these reported findings suggest that, at least within the Ig loci, not only are normal BER, MMR and NHEJ activities altered or re-routed in order to achieve diversification, but that there is uncertainty concerning the respective contributions of DSB repair versus SSB repair to GC activity^19^,^20^.

PC4 is an abundant protein that was originally characterized as a ssDNA-binding protein and general coactivator of transcription^21^,^22^. Its dimeric C-terminal domain crystal structure reveals opposite running ssDNA binding channels^23^, and it has high affinity for ssDNA and heteroduplex DNA^24^. The ssDNA binding activity of PC4 is required to suppress oxidative mutagenesis in *E. coli* and *S. cerevisiae*^17^.

Furthermore, PC4 is recruited to sites of DNA damage independent of γH2AX, has rapid turnover compared to replication protein A (RPA), and PC4 recruitment requires only its C-terminal domain^25^. The C-terminal domain has also been shown to stabilize DNA ends and stimulate end joining, and is not restricted to eukaryotic ligases^26^. Recently, it was reported that the ssDNA binding activity of PC4 is important for genome stability, cell proliferation and mitigation of replicative stress^27^. Mortusewicz *et al*.^27^ showed that PC4 C-terminal-dependent foci form in response to hydroxyurea (HU) treatment, to UV-induced damage in cells pretreated with bromodeoxyuridine and to etoposide treatment, suggesting recruitment to sites of SSBs and DSBs, as well as to replication sites labeled with 5-ethynyl-2′-deoxyuridine prior to HU treatment. Interestingly, they showed that Mre11 inhibition and HU treatment have no effect on PC4 recruitment but do reduce RPA foci formation. They also demonstrated that relative HR activity, as shown by I-SceI induction of a DSB in a DR-GFP reporter, is ~3–5 fold reduced in PC4 knockdown U2OS cells^27^. It was also recently reported that PC4 knockdown impairs the recruitment of non-homologous end-joining factor 1 (XLF) to DSBs in human esophageal squamous cell carcinoma, effectively radio-sensitizing these cells^28^. As our initial observations also suggested that PC4 may play a role in the radiation response (this study), as PC4 has been reported to stimulate NHEJ^26^, and since NHEJ-deficient DT40 cells have significantly higher levels of GC^20^, we tested the role of PC4 in repair of clustered lesions in light chain genes.

## Results

### PC4 status has limited effect on growth rate after irradiation

**Figure 1:**
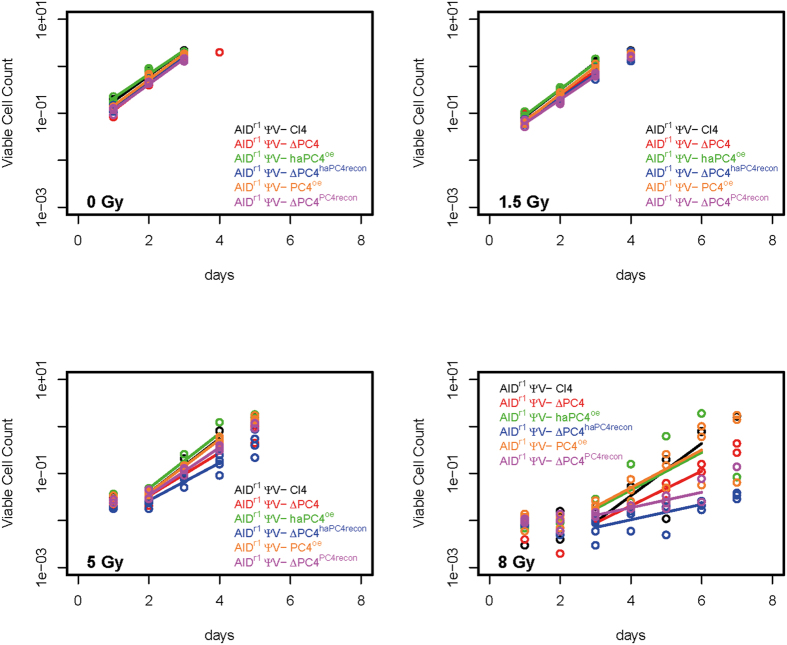
PC4 status has limited effect on growth rate after irradiation. The effects of 0, 1.5, 5 and 8 gray (Gy) on the growth rate of cell lines: AIDr1 ψv- Cl4, AIDr1 ψv- ∆PC4, AIDr1 ψv- PC4^oe^, AIDr1 ψv- haPC4^oe^, AIDr1 ψv- ΔPC4^PC4recon^ and AIDr1 ψv- ΔPC4^haPC4recon^. The bars mark the Log-growth-phase used for analysis.

In a pilot study testing effects of ionizing radiation on various knock-out clones, we identified ∆PC4 as demonstrating increased radiation sensitivity. To further elucidate this finding, we compared the effects of 0, 1.5, 5 and 8 gray (Gy) on the growth rate of the engineered cell lines: AID^r1^ ψv- Cl4 (parental line), ∆PC4 (PC4 knock-out), PC4^oe^ (over-expressing PC4), haPC4^oe^ (over-expressing hemagglutinin-tagged PC4), ΔPC4^PC4recon^ (knock-out reconstituted with PC4) and ΔPC4^haPC4recon^ (knock-out reconstituted with hemagglutinin-tagged PC4). The HA-tagging was performed to facilitate protein co-localization studies (data not shown), but was included here to test for functional phenotype reconstitution and proved to be quite fortuitous. For growth analysis, we tested the parental clone, ∆PC4 and haPC4^oe^ in triplicate, and multiple primary clones of ΔPC4^haPC4recon^ (4× primary clones), PC4^oe^ (3× primary clones) and ΔPC4^PC4recon^ (3× primary clones). Relative to the parental clone, there were insignificant to small statistical differences in the doubling times of ∆PC4 cells at 0, 1.5 and 5 Gy (Fig. 1 and Table 1) demonstrating no general growth defect in the absence of PC4 alone. At 8 Gy, ΔPC4^haPC4recon^ and ΔPC4^PC4recon^ demonstrated significant sensitivity whereas haPC4^oe^ and PC4^oe^ did not. While the doubling time at 8 Gy for ∆PC4 increased by >50% compared to that of the parental clone, the growth variation kept it statistically insignificant. Interestingly, haPC4 exacerbated the knock-out situation with as little as 1.5 Gy, but had no significant effect when overexpressed. This suggests that N-terminal tagging disrupts some activity that was still present when co-expressed with native PC4 and that activity could be partially compensated for in ΔPC4 alone.

Surprisingly, when cloning the bicistronic internal ribosome entry site (IRES) containing PC4iresGFP and haPC4iresGFP expression constructs for random integration into the cells, we encountered what we suspect to be post-transcriptional regulation, including silencing. Once stably integrated into a non-mutating locus, we would expect GFP expression levels to be fairly constant. While we do expect to obtain randomly integrated primary clones showing low or no measureable GFP expression, we did not expect to see differential GFP expression within single-cell-derived clonal populations. We tested 12 primary clones for each, 14 for ΔPC4^haPC4recon^, via flow cytometry and the mean (median) percent GFP reduced, gated for >2-fold reduction in intensity plus negative cells, was 6.9% (1.8%) and 3.4% (0.6%) for haPC4^oe^ and ΔPC4^haPC4recon^, and 15% (7.4%) and 47.2% (45.1%) for PC4^oe^ and ΔPC4^PC4recon^, respectively. This suggests a high degree of regulation of the mRNA transcript, especially for the un-tagged PC4 expression construct and most especially in the ΔPC4 background.

### PC4 is required for HDR in GC-active cells

To investigate the contribution of PC4 to HDR, we engineered a targeted knockout of the gene in the DT40 Cl18 cell line^29^. This cell line harbors an extra guanosine residue in the rearranged Igλ locus, creating a plus-one base frameshift that is predominately repaired via GC. Repair of the frameshift results in the positive surface expression of IgM. The frequency of repair can be monitored by quantifying the surface-IgM (sIgM) status of a clonal population in a reversion assay – sIgM (−) to sIgM (+) –via flow cytometry with fluorescently labelled anti-chicken IgM antibodies. In order to reconstitute the wild type (WT) situation or over-express the protein, as with AID^r1^ ψv- Cl4, we engineered two further DT40 Cl18-based clones using the PC4iresGFP and haPC4iresGFP bicistronic expression constructs. In order to accurately measure and compare GC events, all clones were sorted at the start of the experiment, selecting for IgM (−) in the WT and KO clones and for IgM (−)/GFP (+) in the recon and oe clones. The sorted populations were subcloned to perform a sIgM gain fluctuation analysis on not less than 35 single-cell-derived colonies of each primary clone tested. Additionally, for direct comparison to the fluctuation analysis, DT40 Cl18, DT40 ∆PC4 and DT40 ΔPC4^recon^ were also cultured for two weeks post-sort in bulk for genomic DNA isolation and comparative sequence analysis of the Igλ VJ gene segments.

**Figure 2:**
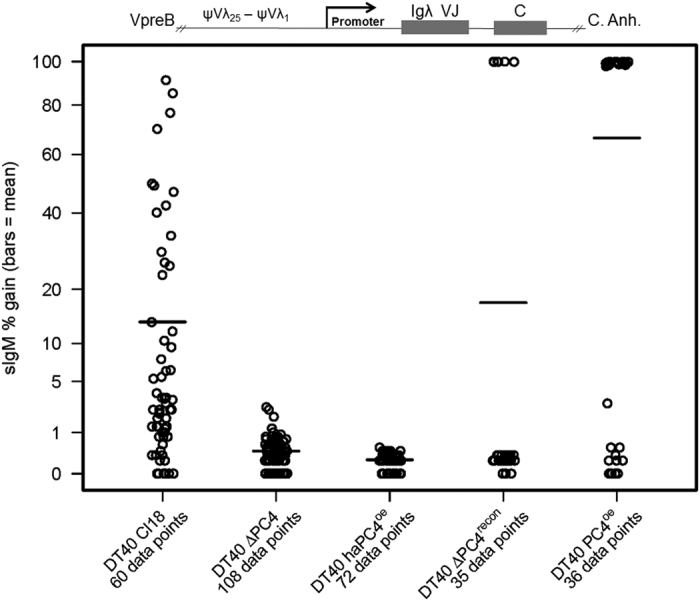
PC4 is required for Gene Conversion. Fluctuation analysis of sIgM reversion assay in GC-active subclones. Cells were sorted for IgM(−) in the DT40 Cl18 (WT) and DT40 ΔPC4 clones, and for IgM(−)/GFP(+) in the DT40 haPC4^oe^, DT40 ΔPC4^recon^ and DT40 PC4^oe^ clones. Following a 48–72 hour post-sort recovery phase, the sorted populations were subcloned by limiting dilution and measured by flow cytometry for % gain of sIgM expression 14 days after sorting. Each subclone population equals one data point and the bar indicates the mean. Loci representation not drawn to scale. Wilcoxon Rank-Sum Test (v. WT): DT40 ΔPC4 & DT40 haPC4^oe^ p = ≤ 0.0001.

The results of the sIgM reversion assay clearly show that disrupting PC4 function significantly reduces GC events (Fig. 2). This phenotype is even more pronounced in the DT40 haPC4^oe^ clones, indicating a dominant negative effect. When trying to reconstitute the WT situation, we experienced difficulty in obtaining viable clones. It took numerous transfection attempts to get primary DT40 ΔPC4^recon^ clones along with additional subcloning of the primary clones to obtain relatively stable transfectants expressing GFP and thus, by inference, PC4. While we easily obtained haPC4^recon^ clones in the AID^r1^ ψv-ΔPC4 line, we were unable to obtain DT40 ΔPC4^haPC4recon^ clones. We suspect a toxicity issue regarding PC4/haPC4 reconstitution in DT40 ∆PC4 cells, as we did not have the same degree of difficulty in obtaining PC4^oe^/haPC4^oe^ transfectants in DT40 Cl18 wild-type cells. Futhermore, the resulting profiles of the sIgM reversion assay of both DT40 PC4^oe^ and DT40 ΔPC4^recon^ indicate that any PC4 activity driving GC took place after sorting for 100% IgM(−) and prior to subcloning for single-cell-derived colonies, and does not appear to be ongoing. This further suggests a high degree of post-translational regulation and silencing of the constitutively expressed mRNA transcript.

**Figure 3:**
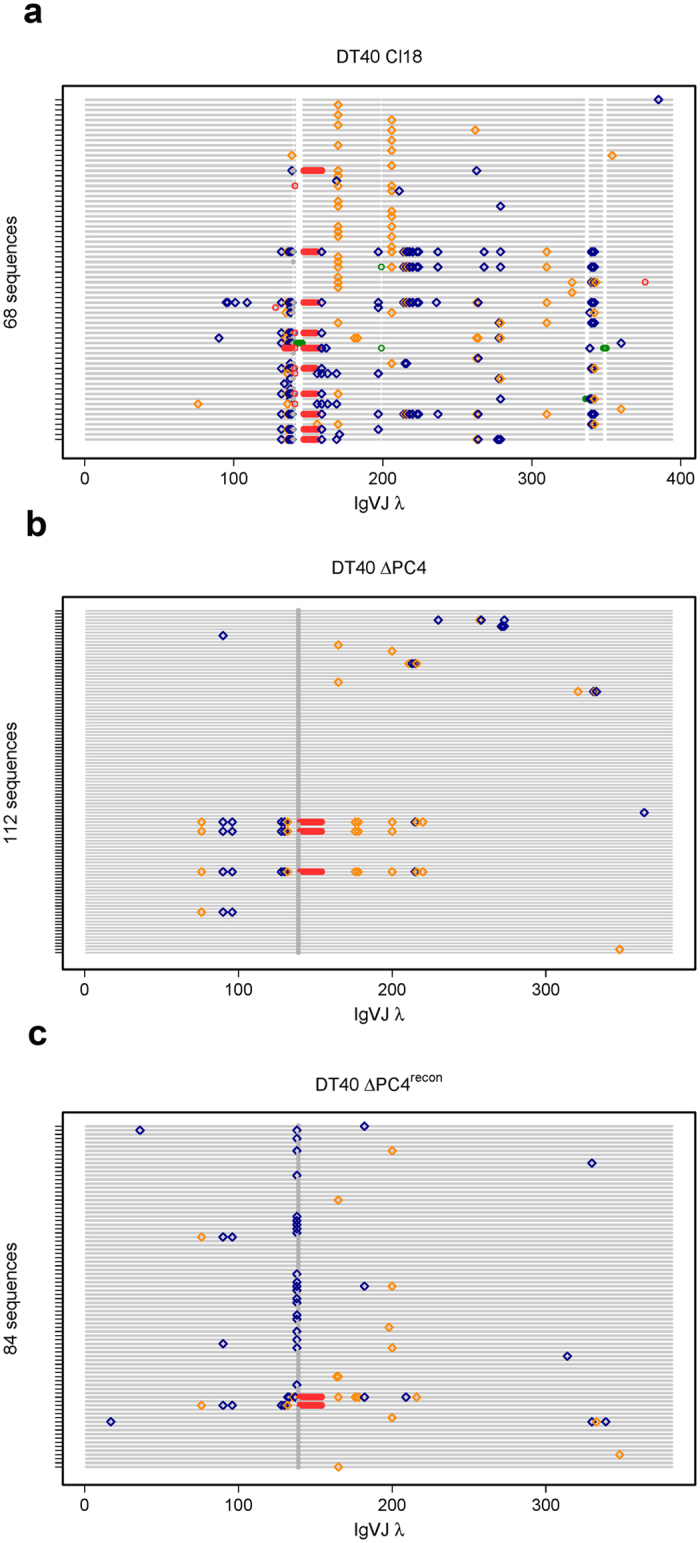
Quick-view mutational analysis reveals PC4’s influence on GC. Quick-view analysis of GC-active bulk population sequences. Cells were sorted for IgM (−) in the DT40 Cl18 (**a**) and DT40 ΔPC4 (**b**) clones and for IgM (−)/GFP (+) in the DT40 ΔPC4^recon^ (**c**) clone. Following 2 weeks bulk growth (~25 generations) of the sorted population, gDNA was recovered from each and the rearranged Igλ VJ segments were sequenced. The Quick-view graphical representation that we use clearly identifies insertions and deletions (green and red circles respectively), transitions and transversions (blue and gold diamonds respectively), and highlights the “+1 G” (grey circle) creating the frameshift.

To investigate PC4’s effects at the DNA level, we compared sequences obtained from the bulk populations of DT40 Cl18, DT40 ∆PC4 and DT40 ∆PC4^recon^ at the same timepoint as the flucuation analysis for direct comparison of the results. While it is impossible to unambiguously distinguish all GC events from all somatic hypermutation (SHM) events in the same sequence, we can plot the results to assemble a trending picture. In our Quick-view analysis (Fig. 3), we see that 84% (57 of 68) of the DT40 Cl18 sequences most likely underwent GC at the frameshift site (defined as loss of the extra guanosine residue) as compared to 2.7% (3 of 112) of the DT40 ∆PC4 cells, and that reconstitution achieved a 27% (23 of 84) rate. Additionally in the DT40 ∆PC4 sequences, we see five “tracts” indicating possible GC events occuring apart from the frameshift site (one upstream and four downstream). While other ψV-templated GC events occurred both at and apart from the frameshift site, the majority of the extra-guanosine-residue removal events in DT40 Cl18 appear most probably to be templated by ψVL8 ~45 of the 57 times, which is in agreement with previous published results^6^. This pseudogene appears to have most probably also templated change ~21 of the 23 times in DT40 ∆PC4^recon^. In DT40 ∆PC4, the three nearly identical sequences with 16 bp deletions at the frameshift site do not match any of the 25 publically available pseudogene sequences. Interestingly, these three sequences match up with high homology to the wild-type DT40 (not Cl18) Igλ sequence (which is missing these 16 bp), as does the second of the two DT40 ∆PC4^recon^ sequences that also contain 16 bp deletions at the frameshift site. Without extensive sequencing of both the >20,000 base pairs upstream (encompassing all 25 pseudogenes) and the complete unrearranged Igλ loci in the cell lines used, we can only speculate as to how this may have come about. However, this pattern is only seen in the sequences of the ∆PC4 cells and in the ∆PC4^recon^ (with questionable PC4 expression) cells.

### PC4 supports SHM in GC-inactive cells

In order to define the effect of PC4 on SHM alone, we used the aforementioned clones of the cell line AID^r1^ ψv- Cl4. This cell line does not undergo GC, due to the absence of pseudogenes, and the AID gene has been knocked out and reconstituted as a bicistronic message attached to a GPT gene (for mycophenolic acid resistance) flanked by LoxP sites^30^. In this cell line model, the rearranged Igλ functions correctly and mutations can be monitored using a sIgM loss assay – sIgM (+) to sIgM (−) – via flow cytometry with fluorescently labelled anti-chicken IgM antibodies. As described previously, all clones were sorted at the start of the experiment, but this time selecting for sIgM (+) in the AID^r1^ ψv- Cl4 precursor and ∆PC4 clones and for IgM (+)/GFP (+) in the haPC4^oe^ and ΔPC4^haPC4recon^ clones. The PC4^oe^ and ΔPC4^recon^ clones were too unstable, as determined by GFP fluorescence or lack thereof, for further testing.

**Figure 4:**
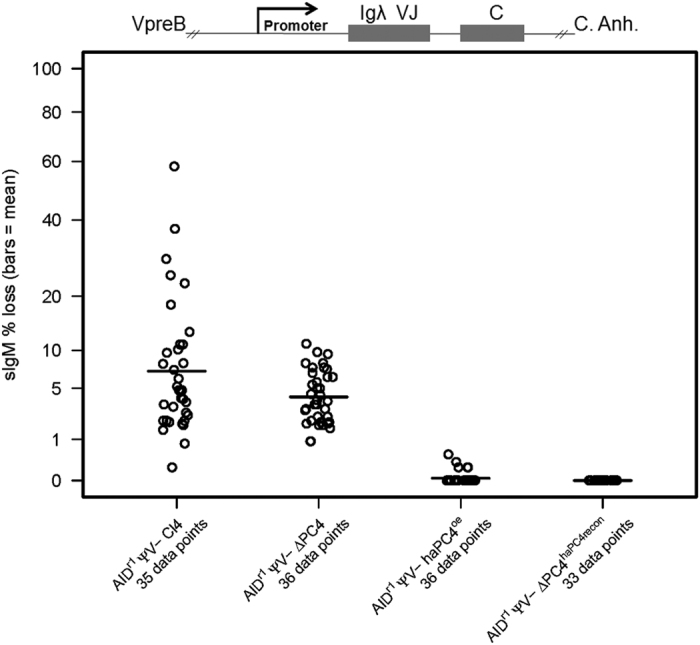
PC4 supports SHM in GC-inactive cells. Fluctuation analysis of sIgM loss assay. Cells were sorted for IgM(+) in the AIDr1 ψv- Cl4 precursor (WT) and ∆PC4 clones, and for IgM(+)/GFP(+) in the haPC4^oe^ and ΔPC4^haPC4recon^ clones. Following a 48–72 hour post-sort recovery phase, the sorted populations were subcloned by limiting dilution and measured by flow cytometry for surface IgM expression 14 days after sorting. Each subclone equals one data point and the bar indicates the mean. Loci representation not drawn to scale. Wilcoxon Rank-Sum Test (v. WT): ΔPC4 p = 0.16; haPC4oe & ΔPC4haPC4recon p = ≤ 0.0001.

**Figure 5:**
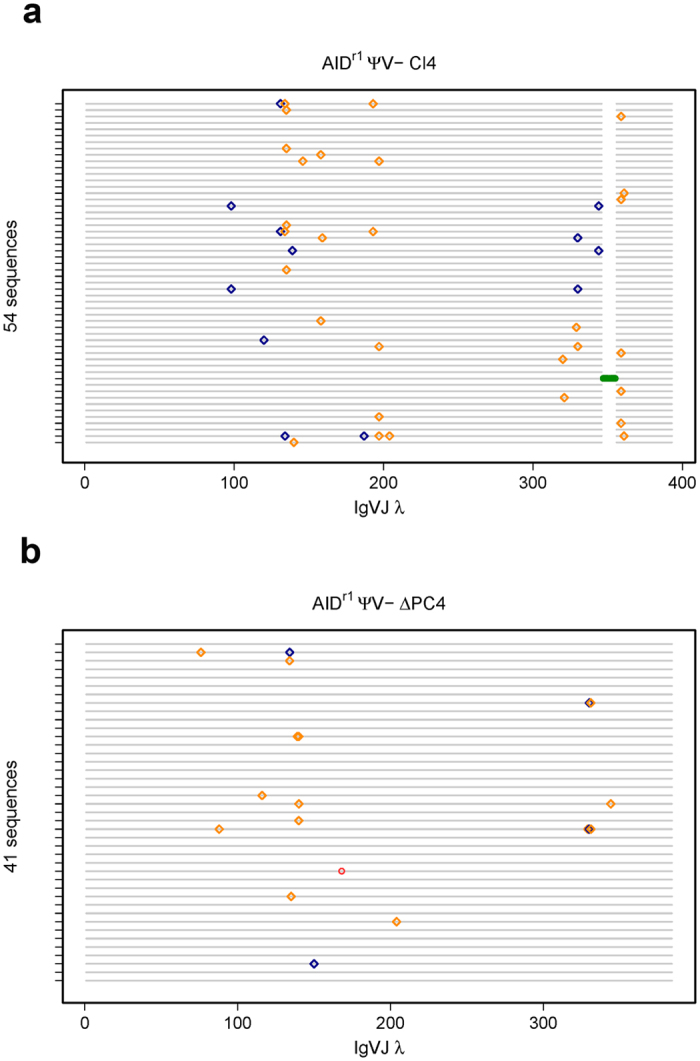
Quick-view mutational analysis reveals PC4’s influence on SHM. Cells were sorted for IgM(+) in the AID^r1^ ψv- Cl4 (**a**) and AID^r1^ ψv- ∆PC4 (**b**) clones. Following two weeks bulk growth of the sorted population, gDNA was recovered from each clone and the rearranged Igλ VJ segments were sequenced for a more complete analysis. Insertions and deletions are green and red circles, respectively. Transitions and transversions are blue and gold diamonds, respectively.

The fluctuation analysis (Fig. 4), clearly shows a > 50% reduction in the sIgM loss rate in the ∆PC4 clone. This is even more pronounced in both the haPC4^oe^ and ΔPC4^haPC4recon^ clones, where sIgM loss is nearly and completely abolished, respectively. We repeated the sequence analysis for these GC-inactive cells to define the role of PC4 at the DNA level absent GC-induced ambiguity. Our Quick-view graph (Fig. 5) shows that not only has the mutation rate dropped, but that the trs:trv ratio has also been altered with the transversion rate being increased from 1:2.3 to 1:3.5 (Supplementary Fig. 1). With the overexpression of the N-terminally hemagglutinin tagged haPC4, the mutation rate is reduced by a factor of 10, and in ΔPC4^haPC4recon^ the single recorded mutation does not deviate from what might be expected to be a procedurally introduced artefact (Supplementary Fig. 1). Our analysis confirms that PC4 promotes SHM and that by presumably disrupting N-terminal activity via the hemagglutinin-tag, normal mutational activity is almost completely blocked.

### PC4 alters the SHM spectrum of a constitutively expressed transgene

**Figure 6:**
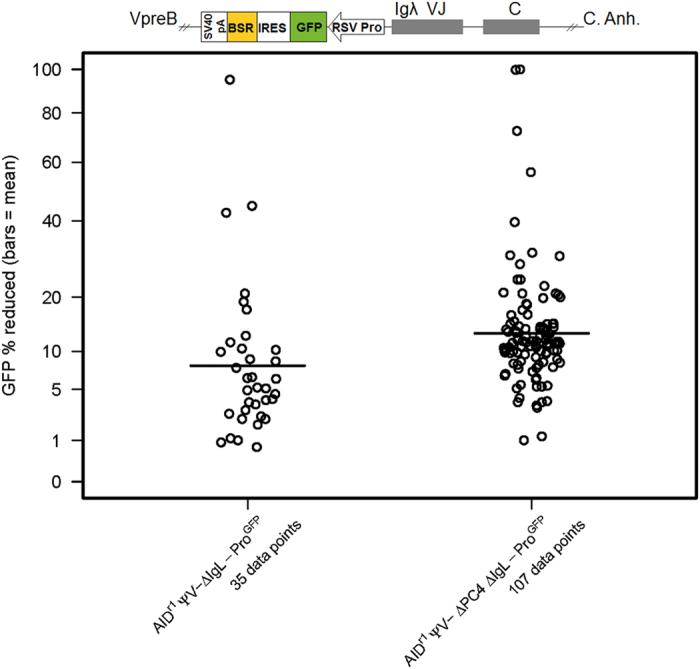
PC4 alters the SHM spectrum of a constitutively expressed transgene (GFP). Fluctuation analysis of GFP intensity loss. Cells were sorted for GFP(+) in the AIDr1 ψv- IgL-Pro−/GFP (WT) and AIDr1 ψv- ΔPC4 IgL-Pro−/GFP clones. Following a 48–72 hour post-sort recovery phase, the sorted populations were subcloned by limiting dilution and measured by flow cytometry for GFP intensity loss 14 days after sorting. Each subclone result contributes one data point and the bar indicates the mean. Loci representation not drawn to scale. Wilcoxon Rank-Sum Test (v. WT): ΔPC4 IgL-Pro−/GFP p = ≤ 0.0001.

**Figure 7:**
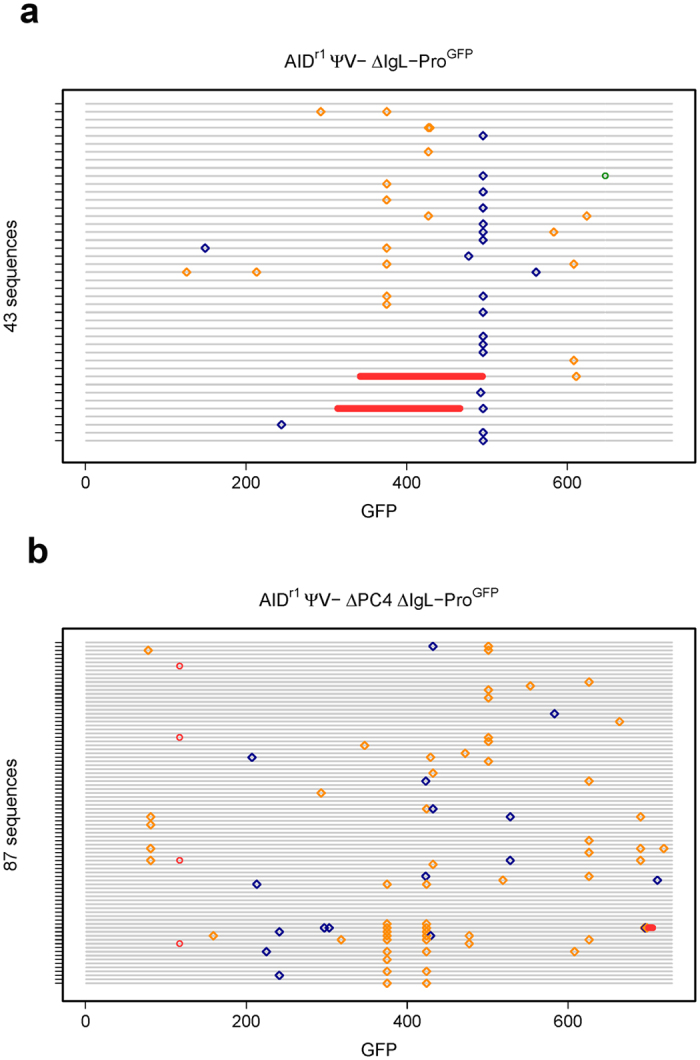
Quick-view mutational analysis reveals PC4’s influence on hypermutation of a constitutively expressed transgene (GFP). Cells were sorted for GFP (+) in the AID^r1^ ψv- IgL-Pro^−/GFP^ (**a**) and AID^r1^ ψv- ΔPC4 IgL-Pro^−/GFP^ (**b**) clones. Following two weeks bulk growth of the sorted population, gDNA was recovered from each and the GFP gene was sequenced for a more complete analysis. Insertions and deletions are displayed as green and red circles, respectively, and transitions and transversions as blue and gold diamonds, respectively.

To test the effect of PC4 on repair in the absence of possible influences of transcriptional regulation, we engineered a targeted knockout of the rearranged Igλ promoter in the cell line AID^r1^ ψv- Cl4 and replaced it with a bicistronic GFPiresBSR construct under a constitutive RSV promoter. In this cell line, the GFP functions properly and the Igλ cis regulatory sequence-induced mutations^31^ lead to loss of GFP intensity that can be monitored via flow cytometry. As described previously, all clones were sorted at the start of the experiment, but this time selecting only for GFP (+). In the fluctuation analysis (Fig. 6), a significant increase in cells with reduced GFP intensity is clearly seen, which at this point was unexpected. However, the Quick-view analysis (Fig. 7) gives a much clearer picture of the apparent increase of mutational activity in the ∆PC4 clone. The mutation rate actually decreased slightly in the ∆PC4 cells, but the number of transversions more than tripled (1:0.95 to 1:3.4) and those occurred predominantly with C**⇔**G (Supplementary Fig. 2). This suggests that in the absence of PC4 and with transcription under control of a constitutive promoter (not cell cycle dependent), REV1 becomes involved, leading to increased C**⇔**G transversions on both strands^32^. This suggests that at the appearance of an abasic site in the pathway, and absent PC4, BER can be rerouted to increased TLS activity, probably as a function of timing and DNA polymerase recruitment^33^.

We also see the first indications, albeit slight, of NHEJ activity with differential deletion patterns that are suggestive of including microhomology-mediated end joining (MMEJ) and resection-induced alternative end joining (A-EJ)^34^. With PC4 present, there are two sequences with very large deletions of 153 bp each and a single sequence with a 1 bp insertion; without PC4, there are five sequences of only short deletions (4 × 1 bp, 1 × 6 bp). These results suggest that alterations in NHEJ outcomes are influenced by PC4 status.

### PC4 status does not alter transcription of AID, Igλ or GFP

To test whether the transcription of the usual suspects (AID, Igλ, GFP transgene) is influenced by PC4 status, we isolated total mRNA from the bulk cultures at the same time-point as we isolated genomic DNA (gDNA). All qPCR replicates were run three or more times (Supplementary Table 1) with the exception of AID expression of the AID^r1^ derived clones where AID has been knocked out and replaced by random ectopic expression. In these, we ran one replicate each of the GFP expressing WT-PC4 and ∆PC4 clones for reference only. As can be readily seen (Supplementary Fig. 3), PC4 status had no discernable effect on the transcription of these three genes.

### PC4iresGFP mRNA levels are sufficient regardless of fluorescent status

To investigate the unexpected variation of GFP fluorescence in cells transfected with the bicistronic messenging contructs, we performed qPCR. We tested DT40 Cl18, DT40 ∆PC4 Cl.5 and freshly sorted GFP positive and GFP negative populations of DT40 PC4^oe^ Cl.11 and DT40 ΔPC4^PC4recon^ Cl.12. All qPCR replicates were run three or more times (Supplementary Table 1). As can readily be seen (Supplementary Fig. 4), PC4 is overexpressed in the transfected clones at the same level as GFP and that level is not diminished in the cells sorted for “GFP negative”. These data further support our postulation of post-transcriptional regulation leading to gene silencing. We would like to note here that the recent publication^28^ reporting full-length PC4 ectopic expression with no mention of difficulty did so by changing the mRNA transcript in order to avoid being targeted by either of their miRNA constructs.

### PC4 status influences early γH2AX foci dynamics but not global DSB repair

To rule out a general DSB repair deficiency, we measured IR-induced γH2AX formation and determined the number of Radiation-Induced γH2AX Foci (RIF). It is evident that irradiation induced DSBs and the associated focus-like phosphorylation of H2AX histones in all conditions (Supplementary Fig. 5). PC4 over-expressing cells (PC4oe and haPC4oe) displayed reduced foci numbers 0.5 h after high dose irradiation at 8 Gy and PC4^oe^ cells also showed reduced foci numbers 4 h after lower dose irradiation at 1 Gy. However, global DSB repair was obviously not compromised, since RIF were resolved 16 h after irradiation in all clones. These results suggest that while PC4 influences immediate to short-term foci dynamics, there is no global double-strand break repair defect absent PC4 in DT40 cells under these conditions.

### PC4 is not required for targeted homologous recombination in DT40 cells

To rule out a general HR deficiency, we tested the targeting efficiency of WT and ∆PC4 cells using constructs targeting the Igλ and AID loci. Surprisingly, the targeting of the Igλ loci showed no defect, whereas GC was significantly disrupted in the same ∆PC4 cell line (Table 2 and Fig. 2). With these results, we conclude there is no general ∆PC4-induced targeted HR deficiency. Furthermore, we speculate that the fact that the targeting constructs already have DSBs (linearized plasmids), unlike the GC situation, could play a role in why targeted HR is not interrupted in the absence of PC4.

## Discussion

Our study began as a test of the role of PC4 in cellular radiation response. During the growth experiments, we failed to observe statistically significant differences in the cell doubling times between the WT and the PC4 knock-out cells in the different conditions tested. However, the reconstitution of ∆PC4 cells with both ha-tagged and untagged PC4 made them significantly more sensitive to irradiation as measured by their doubling rates. Additionally, γH2AX radiation-induced foci analysis suggested an early effect on focus response with over-expression of PC4 after irradiation at 1 and 8 Gy. Eventually radiation-induced DSB foci were resolved 16 h after irradiation in all clones tested, suggesting no general defect in global DSB repair under these conditions. These results suggest to us that the suspected role of PC4 in DNA repair is more complex than previously reported and, at least in our cell model, less likely to be due to transcriptional aspects associated with PC4.

Since the discovery of AID and its role in the immune response^1^, much emphasis has been placed on how simple deamination events can lead to such diversity. Many proteins known to be involved in various pathways of faithful DNA repair have been shown to also be involved in aspects of immunoglobulin “repair” leading to diversification. This apparent paradox has led to speculation concerning faithful DNA repair being coopted by locus-specific error-prone repair actors. However, the mechanism leading from the relatively simple repair of a deaminated base to a situation resulting in the recruitment of, for example, error-prone polymerases is poorly understood. Even the most comprehensive mechanistic model for uracil processing outcomes in the context of class-switch recombination and somatic hypermutation, the Neuberger model^4^,^35^,^36^, does not answer the fundamental question of why normally error-free BER gets re-routed in the first place.

**Figure 8:**
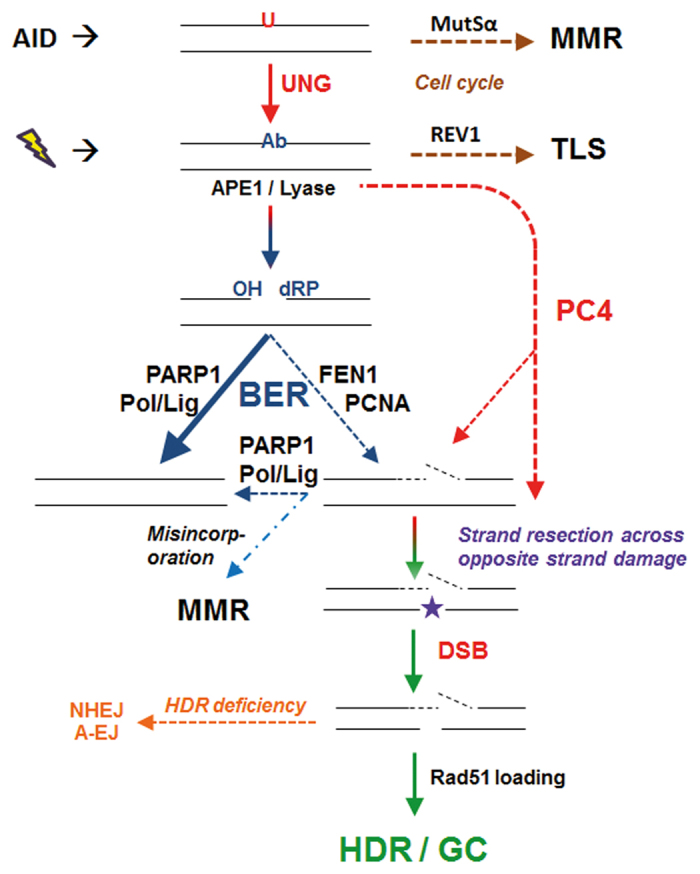
Proposed model of the role of PC4 in DNA repair re-routing. PC4 alters the dynamics of abasic site repair. In the context of clustered lesions, this can lead to staggered DSBs requiring HR/EJ repair.

Here, we show that PC4 has a role in answering that question, as well as influencing DNA repair outcome. In our working model based on previous studies and the data presented here (Fig. 8), PC4 appears to be involved at the point of APE1/lyase recruitment/activity. Delaying this step or creating abasic sites later in the cell cycle could result in increased replicative TLS (cell cycle induction of damage tolerance), which is suggested by our results with AID^r1^ ψv- ΔPC4 Igλ-Pro^−/GFP^ where transcription of GFP is under control of a heterologous promoter. What we do not see is any significant increase in transition mutations, specifically C→T, to suggest involvement at the point of or upstream of UNG activity. At this point in our model, normal short-patch BER would predominate at a simple abasic site 80–90% of the time, with repair leaving no trace behind^37^. Our data suggest that PC4 plays a role in influencing the short−/long-patch ratio of BER leading to an increased chance of a GC event. This could come about by directly or indirectly promoting long(er)-patch DNA repair or something NER-like. In areas of lesion clusters, this could lead to increased staggered DSBs, shifting the dynamics of repair and path choice. This is likely further influenced by cell cycle timing, extent of damage and availability of homologous sequences. In agreement, we see trace evidence for NHEJ when homologous donor sequences (pseudo-genes) are absent in the rearranged locus, as with AID^r1^ ψv- Cl4, with a single small 9 bp insertion in the WT clone and a single 1 bp deletion in the ΔPC4 clone. However, we see more convincing evidence for end-joining repair activity in the clones harboring the GFP transgene under a constitutive promoter. With homologous sequences present, PC4 promotes HDR; absent homologous sequences, PC4 appears to influence NHEJ outcomes. The latter is most likely a function of PC4’s C-terminal DNA binding domain and end-tethering^26^. In addition, the short-patch/long-patch BER ratio appears to be more as expected absent PC4 and thus the incidence of repair-induced DSB is much lower. We see this in the occasional induction of imprecise NHEJ (5 possible events out of 87 sequences) in AID^r1^ ψv- ΔPC4 Igλ-Pro^−/GFP^ that is comparable to the frequency of GC events in DT40 ΔPC4 (8 possible events out of 112 sequences).

PC4 is a transcriptional coactivator, but represses transcription in the absence of the basal transcription factor II H (TFIIH)^38^, which is also implicated in excision repair^39^. XPB is a 3′→ 5′ helicase subunit of TFIIH that interacts directly with PC4^40^ and is essential for the repair and transcription activity of TFIIH^41^. In humans, both PC4 and XPB interact with p53 for activation and function, with PC4 possessing the ability to bend DNA and XPB promoting the unwinding of base pairs^42^,^43^. However, p53 is disrupted in DT40 cells and thus plays no role^44^. Recently, a putative bacteriophage homolog of PC4 was identified through sequence homology and crystal structure, and it is thought to play an essential role in recombination-dependent DNA replication^45^. This suggests a high degree of evolutionary conservation. Taken together, PC4 seems to participate in and monitor transcription/replication, and moreover is involved in the response to DNA damage during these processes. It interacts with other key proteins of the DDR at a very early stage to control the decision of repair pathway choice. Our data point to PC4 activity being recruited to this decision step at the point of the creation of an abasic site (Fig. 8). Our data further suggest that by N-terminally tagging PC4 with HA, we dominantly interrupt an uncharacterized activity of PC4 that is crucial to normal function. We suspect that this uncharacterized damage response activity is associated with DNA capping/uncapping and/or resection dynamics that becomes determinative in the context of lesion clusters. While we don’t suspect it, we cannot conclusively exclude that haPC4’s dominant negative effect on GC and SHM is simply a function of blocking or retarding AID activity. Furthermore, we cannot rule out that the N-terminal tag blocks a co-factor or recruitment function associated with, for example, APE1, 53BP1, PARP1 or even MRE11. This aspect will be the subject of further investigation.

We conclude that PC4 is crucial to early DNA repair pathway re-routing within the context of clustered lesions requiring a cluster fix, as in the case after exposure to high doses of ionizing radiation (clustered complex lesions) or AID-induced (multiple abasic sites) like in the immune response.

## Materials and Methods

### Cell culture

DT40 clones used were cultured in Chicken Medium (CM; DMEM/F-12 supplemented with 10% fetal bovine serum, 1% chicken serum, 2 mM L-glutamine, 0.1 mM β-mercaptoethanol and penicillin/streptomycin) at 41 °C in a 5% CO_2_ environment. To maintain AID expression in the AID^r1^ ψv- Cl4 based clones, cells were cultured occassionally in the presence of 0.5 mg/ml of mycophenolic acid as previously described^46^.

### Knockout, knock-in and transfections

The creation of a knockout targeting construct and transfections were as previously described^47^,^48^. Supplementary Table 2 lists the primers used. Briefly, the upstream and downstream targeting arms for the PC4 (single allele on Chromosome Z) knockout construct were created using the primer pairs rbc132-rbc455 and rbc456-rbc137 respectively. This targeted the insertion of two in-frame stop codons (K3amber, S4ochre) and deleted the rest of exon 1, the first intron and exon 2. The deleted genomic sequence was replaced with a BamHI-restricted selectable marker cassette harboring the floxed gene for blasticidin (Bsr) or puromycin (Puro) resistance under the control of a CAG promoter. Targeting confirmation was done via PCR using the primer pair rbc131-BS1 (BSR) or rbc131-PU5 (Puro) and knockout confirmation via PCR using the primer pair rbc503-rbc504 targeting a segment of the deleted sequence.

For the creation of PC4 knock-in overexpression (oe)/reconstitution (recon) variants, we used the PC4 cDNA-harboring plasmid 12p22^49^ for the PCR template. For untagged PC4 oe/recon, the primer pair rbc736-rbc672 was used and for the HA/ha (hemagglutinin) tagged - haPC4 - expression, the primer pair rbc671-rbc672 was used. The HA tag DNA used produced the amino acid sequence YPYDVPDYA and was inserted via PCR immediately after the methionine start codon. The PCR products were cloned into a pBluescriptKS+ (Agilent Technologies) backbone harboring a pCAG-(multi-cloning site)-ires-GFP-CAG-Puro expression construct using the restriction enzymes NheI and BglII. This construct allows for random integration of the transgene and qualitative monitoring of expression levels via GFP.

For the cloning of the fluorescent protein transgene into the Igλ locus for the GFP loss assay, we used our ForGene plasmid system (pFG). Briefly, the BamHI-restricted basic FG cassette is an RSV viral promoter followed by a bicistronic messaging system consisting of a modified NheI-EcoRV-BglII multi-cloning site, IRES-BSR selection and an SV40 poly-A signal sequence, also in a pBluescriptKS+ backbone. The GFP was cloned into the ForGene variant plasmid pFG2 (pFG2GFP), targeting replacement of the rearranged Igλ-Promoter as previously described^31^, creating transfectants that are directionally transcribed opposite that of normal Igλ function. For an over-view of clones tested/generated and the experiments each were used in, see Supplementary Table 1.

For testing of targeting efficiency (homologous recombination), in addition to pFG2GFP (described above), targeting constructs were created harboring a GFP-NTR fusion reporter for the AID locus as previously described^31^,^47^,^48^. Briefly, AID^r1^ ψv- Cl4, AID^r1^ ψv-∆PC4 Cl3, DT40 Cl18 and DT40 ∆PC4 Cl5 cells were cultured to >80% viability, transfected with the selected NotI-linearized targeting construct as indicated in Supplementary Table 2 and evaluated for targeting efficiency via PCR.

### Growth rates post irradiation

Primary clones tested were grown to >80% viability (Vi-CELL, Beckman Coulter) and had 10^7^ viable cells harvested for each experiment. The cells were gently spun down, resuspended in 10 ml CM and placed in a T25 cell culture flask. The cell containing flasks were irradiated with 0 (sham), 1.5, 5 and 8 Gy using a HWM D-2000 closed unit irradiation chamber (^137^Cs, 0.51 Gy/min). After irradiation, 2 × 10^6^ viable cells were inoculated into a T75 culture flask with a total volume of 50 ml CM for a starting concentration of 0.04 × 10^6^ cells/ml. Growth and viability were measured at 24 hour intervals until overgrowth or for up to 9 days.

### Radiation induced foci (RIF)

Cells were irradiated at room temperature with 240 kV X-rays filtered with 3 mm beryllium at a dose rate of 1 Gy/min at 13 mA (YXLON Maxishot, Hamburg, Germany). Absorbed dose was measured with a PTW Unidos dosimeter (PTW Freiburg GmbH, Germany). Control cells were sham-irradiated. After repair incubation for 0.5, 4 and 16 h, cells were washed two times with PBS followed by fixation in 70% ethanol and stored at −20 °C until further use^50^. For γH2AX foci staining, cells were centrifuged onto glass slides using a Shandon Cytospin 3 Cytocentrifuge (Thermo Scientific). After extraction with 0.25% TX100 in PBS for 20 min, cells were stained with mouse anti-γ-H2AX antibodies (Millipore) at 1:250 in a moist chamber at 37 °C for 1 h. After three 5 min washes in PBTG (PBS, 0,1% BSA, 0.05% Tween20 and 0.5% fish gelatin (Sigma-Aldrich)), primary antibodies were detected with secondary goat anti-mouse Alexa-488 Abs (1:500 in PBTG) for 45 min, followed by three 5 min washes in PBTG. After embedding in Roti-Mount (Carl Roth) anti-fade solution, the number of irradiation-induced DNA damage and repair protein foci was analyzed using a dedicated γH2AX foci classifier for the automated Metafer4 image analysis system (MetaSystems). At least 500 (usually 700) intact and non-overlapping nuclei were analyzed per sample. Three to four independent staining experiments/condition were analyzed for each clone. RIF were obtained by subtracting the values of the corresponding non-irradiated cells from those of the irradiated cells. The average values of the different time points were derived from 3–4 experiments per condition.

### Flow cytometry

For the monitoring and sorting of cells based on IgM status, cells were labelled with the primary mouse anti-chicken IgM-UNLB antibody (0.5 mg/ml, Southern Biotech, Alabama) followed by secondary antibody labelling with goat anti-mouse IgG, human ads-PE (0.5 mg/ml, Southern Biotech, Alabama). Briefly, cells were resuspended in labelling buffer (10% FBS, 0.005% sodium azide in sterile PBS) containing the 1° antibody at 1:500 dilution and incubated on ice for 30 min. The cells were washed with sterile PBS and then resuspended in labelling buffer containing the 2° antibody at 1:100 dilution and incubated in the dark on ice for 30 min. The cells were given a final wash in sterile PBS and resuspended in sterile PBS for sorting and/or testing by flow cytometry. For monitoring GFP status alone, cells were washed twice and resuspended using sterile PBS.

Flow cytometry was performed using the LSR II (BD Biosciences). Briefly, 20,000 cells were counted for each subclone using the 488 nm filter for excitation and measuring either GFP at 530 nm, Phycoerythrin (PE) at 575 nm or both with appropriate compensation. For both gain and loss measurements, positive and negative controls were used to set the appropriate gating. Gating was stringently maintained to avoid capturing dead auto-fluorescing cells or normal cloud fluctuation. Fluorescent activated cell sorts were performed using the FACSStar PLUS (BD Biosciences). Briefly, 200,000 cells were sorted into CM based on the parameters for GFP and/or PE as above. The cells were cultured in 6-well plates for a 48–72 hour post-sort-recovery phase. Following recovery, the cells were used to obtain single cell-derived sub-clones by limiting dilution with the remainder used to seed bulk cultures.

### PCR, cloning and sequencing

For sequencing of the Igλ VJ segments and the GFP transgene two weeks post sort (25 generations based on ~13 hour doubling-time), genomic DNA (gDNA) was isolated from the bulk cultured cells using the DNeasy Blood & Tissue Kit (Qiagen, Germany) according to the manufacturer’s instructions. The gDNA was then used as template for the primer pair rbc765-rbc767 (Igλ VJ) and rbc583-rbc587 (GFP) as previously described^46^ using Pfu Ultra DNA Polymerase (Agilent Technologies). The PCR products were prepared for cloning using EcoRI (New England Biolabs) and ApaI (Thermo Scientific), gel isolated (GeneJET Gel Extraction Kit, Thermo Scientific) and cloned (Takara Ligation Kit, Clontech) into pBluescriptKS+ (Agilent Technologies) for blue-white colony selection, all according to manufacturer’s instructions. Sequencing was done using the BigDye Terminator v3.1 Cycle Sequencing Kit (Thermo Fisher Scientific) with primers rbc764 (Igλ VJ), and rbc734 and rbc585 (GFP), and with the protocol modified as previously described^46^. As mutations are an ongoing process, the parental sequences used for mutational analysis with clonal changes specified are listed in Supplementary Table 3.

### Quantitative PCR

To ensure transcription levels of key genes were not influenced by PC4 status, cDNA was made from total mRNA using the RNeasy Mini Kit (Qiagen) and SuperScript III First-Strand Synthesis System (Thermo Fisher Scientific) according to manufacturer’s instructions. Quantitative PCR (qPCR) was performed on the LightCycler 1.5 (Roche) using the LightCycler FastStart DNA Master SYBR Green I (Roche) kit according to manufacturer’s instructions. Primers used were rbc9-rbc10 for the B-cell activating factor (BAFF) control, rbc22-rbc4 for Igλ, AI24-AI25 for AID, and rbc732-rbc585 and rbc265-rbc585 for GFP.

To evaluate PC4iresGFP transcript stability, cDNA was made as above from bulk or GFP sorted (positive and negative) populations, however the qPCR was performed on the ViiA 7 System (Applied Biosystems) using the SYBR Advantage qPCR Premix (Clontech) according to manufacturer’s instructions. Primers used were rbc9-rbc10 for the BAFF, AI24-AI25 for AID, rbc615-rbc616 for PC4 and rbc843-rbc844 for GFP.

### Statistical analysis

For testing differential growth rates between WT and genetically altered clones (Fig. 1 and Table 1), log-linear models were fitted to the data within the growth phase marked. Significance was determined by the ANOVA F-test, and p-values less than 0.05 were considered as significant.

For comparison between WT and the genetically altered clones (Figs 2,4 and 6) in the fluctuation analyses, the Wilcoxon rank test was used for the comparison of the subclone data points, and p-values less than 0.05 were considered as significant.

For the Radiation-Induced γH2AX Foci Analysis, the Student’s *t*-test was applied to the RIF/cell differences between the replicates tested at a given timepoint and p-values less than 0.05 were considered as significant.

## Additional Information

**How to cite this article**: Caldwell, R. B. *et al*. Positive Cofactor 4 (PC4) is critical for DNA repair pathway re-routing in DT40 cells. *Sci. Rep.* **6**, 28890; doi: 10.1038/srep28890 (2016).

## Supplementary Material

Supplementary Information

## Figures and Tables

**Table 1:** Growth rate (doubling time) of actively growing clones post-irradiation and their p-values compared to AIDr1 ψv- Cl4 (WT).

Clones	Growth Rate (doubling time in hours)				F-test, ANOVA for growth rate coefficient comparison (P-values)			
	0 Gy	1.5 Gy	5 Gy	8 Gy	0 Gy	1.5 Gy	5 Gy	8 Gy
**AID**^**r1**^ **ψv- Cl4**	13.3	12.5	12.5	13.0	NA	NA	NA	NA
**AID**^**r1**^ **ψv- ∆PC4**	12.4	13.7	15.7	19.7	0.526	0.222	0.116	0.191
**AID**^**r1**^ **ψv- haPC4**^**oe**^	14.2	12.9	12.3	18.1	0.535	0.580	0.927	0.461
**AID**^**r1**^ **ψv- ΔPC4**^**haPC4recon**^	13.7	14.2	18.0	44.3	0.696	0.050	0.025	0.006
**AID**^**r1**^ **ψv- PC4**^**oe**^	13.1	12.4	12.7	18.0	0.881	0.898	0.838	0.349
**AID**^**r1**^ **ψv- ΔPC4**^**PC4recon**^	13.3	14.2	14.2	44.3	0.980	0.043	0.267	0.012

**Table 2:** Targeting efficiency in WT and ∆PC4 cell lines.

Parental Line	Cell Viability	Targeting Construct		Efficiency			
	(%)	AID-pGFPNTR	IgλPromoter-pFG2GFP	# Colonies	# Targeted	%	Combined
**AID**^**r1**^ **ψV- Cl4**	**93.7%**	1x		31	31	**100.0**	**100%**
	**82.6%**	1x		9	9	**100.0**	
**AID**^**r1**^ **ψV- ∆PC4 Cl3 (BSR)**	**89.9%**	1x		18	18	**100.0**	**100%**
	**88.2%**	1x		7	7	**100.0**	
**DT40 Cl18**	**87.1%**		1x	51	48	**94.1**	**91%**
			1x	28	24	**85.7**	
**DT40 ∆PC4 Cl5 (Puro)**	**91.8%**		1x	41	41	**100.0**	**98%**
			1x	42	40	**95.2**	
