# Supplementary material for: Positive Cofactor 4 (PC4) is critical for DNA repair pathway re-routing in DT40 cells
Source: Sci Rep. 2016 Jul 4;6:28890. doi: 10.1038/srep28890 (PMC4931448; doi:10.1038/srep28890)
Supplement: Supplementary Information [file srep28890-s1.doc]

**Supplemental to: Positive Cofactor 4 (PC4) is critical for DNA repair pathway re-routing in DT40 cells**

Randolph B Caldwell^*^, Herbert Braselmann, Ulrike Schötz, Steffen Heuer, Harry Scherthan, and Horst Zitzelsberger

^*^Corresponding author:

**Randolph B Caldwell**

Helmholtz Center Munich-German Research Center for Environmental Health (GmbH)

Ingolstädter Landstr. 1, 85764 Neuherberg, Germany

Tel: +49 89 31872621, Email: randolph.caldwell@helmholtz-muenchen.de

**Supplementary Table 1. Over-view of clones used and experiments performed**


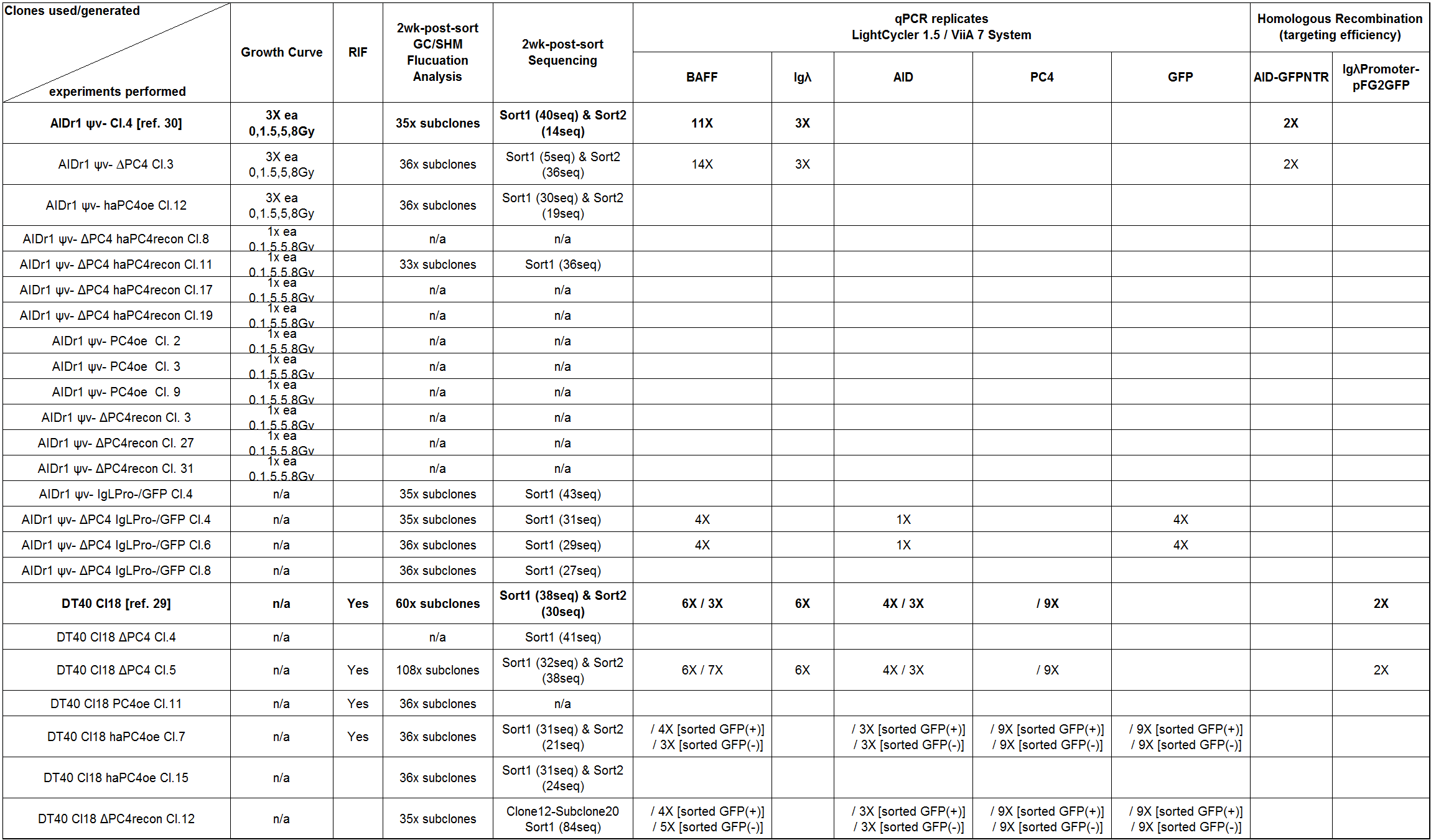


**Supplementary Table 2. Primers used**

| BS1 | cgattgaagaactcattccactcaaatataccc |
| --- | --- |
| PU5 | cccaccgactctagaggatcataatcagcc |
| AI24 | cccagatcttgcttgtgaagtcttcttattgctg |
| AI25 | cccgctagcgccaccatggacagcctcttgatgaagagga |
| rbc4 | atgtcacaatttcacgatgg |
| rbc9 | aactctccctctggctttag |
| rbc10 | agacacttgcaggaaggaag |
| rbc22 | gagtcgctgacctcgtctcg |
| rbc131 | gggctcgaggtcgacctgttcccggcctgtctttctctcc |
| rbc132 | gggctcgaggtcgacctcctctccgtcgcctcgtcttcgc |
| rbc133 | gaagatctggatccctagctcgaagacacgagttcctttgac |
| rbc135 | gaagatctggatccaggcagcctgttacatcatattcag |
| rbc137 | gggtctagaactagtctgccacgggcaggttcaccaatca |
| rbc265 | gatcactctcggcatggacga |
| rbc503 | gaggagggagaagatcttggtatcagctgcccc |
| rbc504 | cagtagaggccacacactactaccatgcaacacgc |
| rbc585 | tatagacaaacgcacaccggcctta |
| rbc615 | cgtgtcttcgagctcatctg |
| rbc616 | catctctgttgctgctttgc |
| rbc671 | actagtgctagcgccaccatgtacccatacgatgttccagattacgctcctaagtcaaaggaactcgtgtcttcgagctca |
| rbc672 | ggatcctctagatctttacagttttcttactgcatcatcaatatcagaaatctgttccttcagc |
| rbc732 | tcacatggtcctgctggagttcgtgac |
| rbc734 | ctagctcgatacaataaacgccatttgaccattca |
| rbc736 | ttactagtgctagcgccaccatgcctaagtcaaaggaactcgtgtcttcgagctcatc |
| rbc764 | tagggcccgggcgtgctgcggaaggacgcgggta |
| rbc765 | gtgggcccagggaggtgtggggagaggcgtaggac |
| rbc767 | tagaattcagcccaggctgccgcgctgagtg |
| rbc843 | ttcttcaagtccgccatgcc |
| rbc844 | aagtcgatgcccttcagctc |
| AID 5'-arm For | gggctcgaggtcatctgagagagagaacccagctgacatgg |
| AID 5'-arm Rev | gggggatccgcttcacaacttaacagaggtaggtttca |
| AID 3'-arm For | gggggatccgtgagagtactgaactgagtcctggacag |
| AID 3'-arm Rev | gggactagtcagtcaacatcaggcaggaagatctggttt |
| AID targeting | gggctcgaggtcatctgagagagaacccagctgacatgg |
| Igλ-Pro targeting | ccagctaattgaggacgatgtgaaagtag |

| **Supplementary Table 3. Parental sequences used for mutational analysis with clone specific changes listed.** | | | | | | | | | |
| --- | --- | --- | --- | --- | --- | --- | --- | --- | --- |
| **Igλ VJ (AIDr1 ψV- Cl4 based clones)** | | | | | | | | | |
| ATGGCCTGGGCTCCTCTCCTCCTGGCGGTGCTCGCCCACACCTCATCCCTGGTGCAGGCAGCGCTGACTCAGCCGGCCTCGGTGTCAGCAAATCCAGGAGAAACCGTCAAGATCACCTGCTCCGGGGGTGGCAGCTATGCTGGAAGTTACTATTATGGCTGGTACCAGCAGAAGTCTCCTGGCAGTGCCCCTGTCACTGTGATCTATGACAACGACAAGAGACCCTCGGACATCCCTTCACGATTCTCCGGTTCCAAATCCGGCTCCACAGCCACATTAACCATCACTGGGGTCCGAGCCGATGACGAGGCTGTCTATTTCTGTGGGAGCTACGAAGACAACAGTGGTGCTGCATTTGGGGCCGGGACAACCCTGACCGTCCTA | | | | | | | | | |
| **AID^r1^ ψV- Cl4** | no change | |  |  |  |  |  |  |  |
| **AID^r1^ ψV- ∆PC4** | C235A |  |  |  |  |  |  |  |  |
| **AID^r1^ ψV- haPC4^oe^** | no change | |  |  |  |  |  |  |  |
| **AID^r1^ ψV- ∆PC4^haPC4recon^** | C235A |  |  |  |  |  |  |  |  |
|  | | | | | | | | | |
| **eGFP** | | | | | | | | | |
| ATGGTGAGCAAGGGCGAGGAGCTGTTCACCGGGGTGGTGCCCATCCTGGTCGAGCTGGACGGCGACGTAAACGGCCACAAGTTCAGCGTGTCCGGCGAGGGCGAGGGCGATGCCACCTACGGCAAGCTGACCCTGAAGTTCATCTGCACCACCGGCAAGCTGCCCGTGCCCTGGCCCACCCTCGTGACCACCCTGACCTACGGCGTGCAGTGCTTCAGCCGCTACCCCGACCACATGAAGCAGCACGACTTCTTCAAGTCCGCCATGCCCGAAGGCTACGTCCAGGAGCGCACCATCTTCTTCAAGGACGACGGCAACTACAAGACCCGCGCCGAGGTGAAGTTCAAGGGCGACACCCTGGTGAACCGCATCGACCTGAAGGGCATCGACTTCAAGGAGGACGGCAACATCCTGGGGCACAAGCTGGAGTACAACTACAACAGCCACAACGTCTATATCATGGCCGACAAGCAGAAGAACGGCATCAAGGTGAACTTCAAGATCCGCCACAACATCGAGGACGGCAGCGTGCAGCTCGCCGACCACTACCAGCAGAACACCCCCATCGGCGACGGCCCCGTGCTGCTGCCCGACAACCACTACCTGAGCACCCAGTCCGCCCTGAGCAAAGACCCCAACGAGAAGCGCGATCACATGGTCCTGCTGGAGTTCGTGACCGCCGCCGGGATCACTCTCGGCATGGACGAGCTGTACAAGTGCGGACTCTAA | | | | | | | | | |
| **AID^r1^ ψV- ∆IgL-Pro^GFP^** | no change | |  |  |  |  |  |  |  |
| **AID^r1^ ψV- ∆PC4 ∆IgL-Pro^GFP^** | Clone 4 | C375G | G501C |  |  |  |  |  |  |
|  | Clone 6 | C375G |  |  |  |  |  |  |  |
|  | Clone 8 | C424G |  |  |  |  |  |  |  |

**Supplementary Fig 1**

**a b**

**
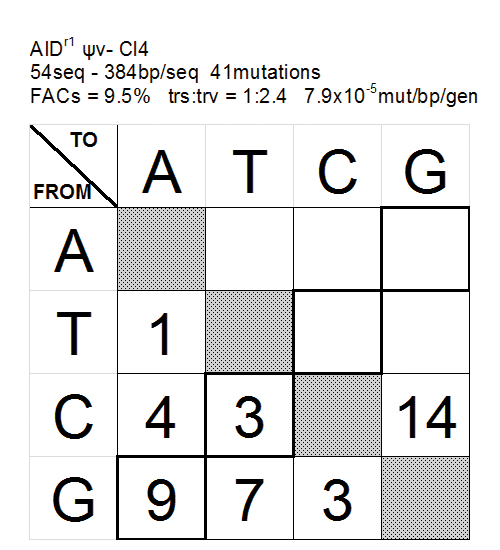

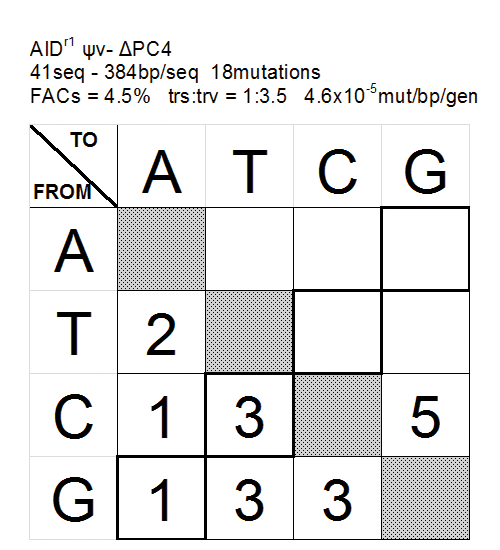
**

**c d**

**
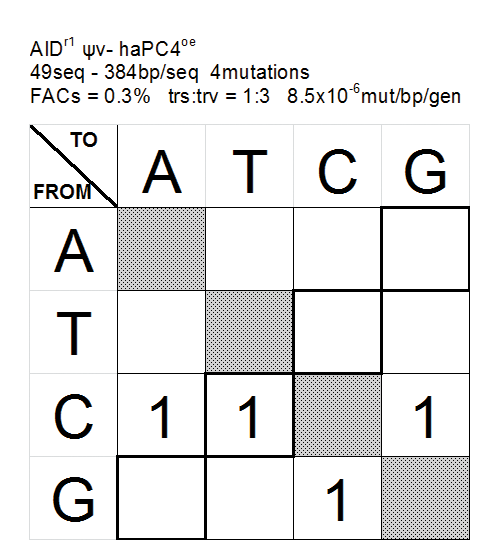

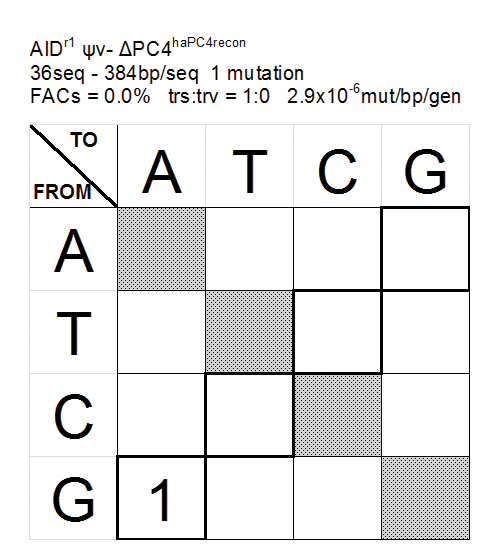
**

**Mutation analysis of Igλ VJ segments in bulk population of GC-inactive cells.**

Results of AIDr1 ψv- precursor (a), ∆PC4 (b), haPC4^oe^ (c) and ΔPC4^haPC4recon^ (d) clones detailing number of sequences (seq), base pairs (bp), mutations (mut) and mean flow cytometry (FACs) results two weeks post sort or 25 generations (gen) based on a doubling time growth rate of ~13 hours. The bold-outlined diagonal boxes from lower left to upper right indicate transition (trs) changes, all others are transversions (trv).

**Supplementary Fig 2**

**a b**

**
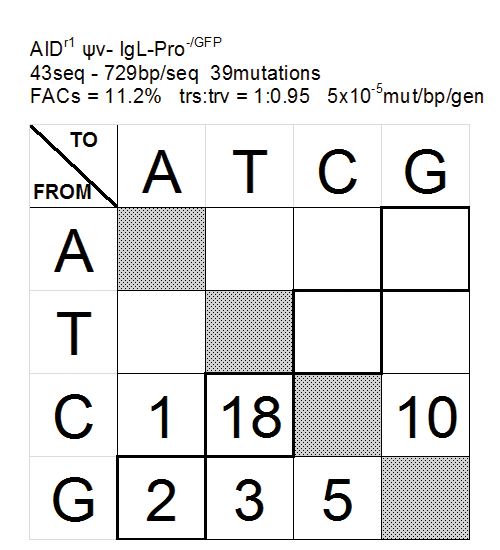

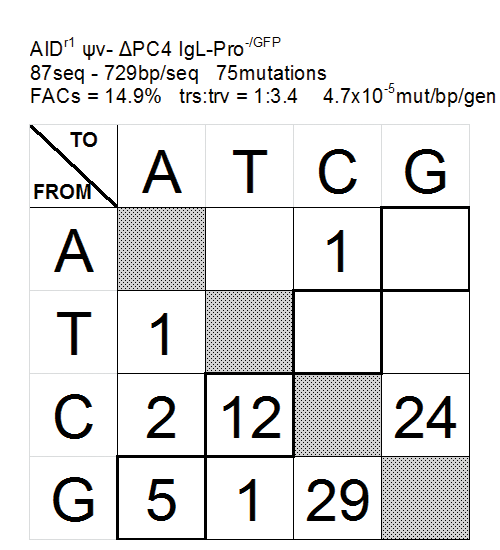
**

**Mutation analysis of GFP in bulk populations.**

Results of AIDr1 ψv- IgL-Pro-/GFP (a) and AIDr1 ψv- ΔPC4 IgL-Pro-/GFP (b) clones. See Supplementary Fig. 1 legend for abbreviations.

**Supplementary Fig 3**

**
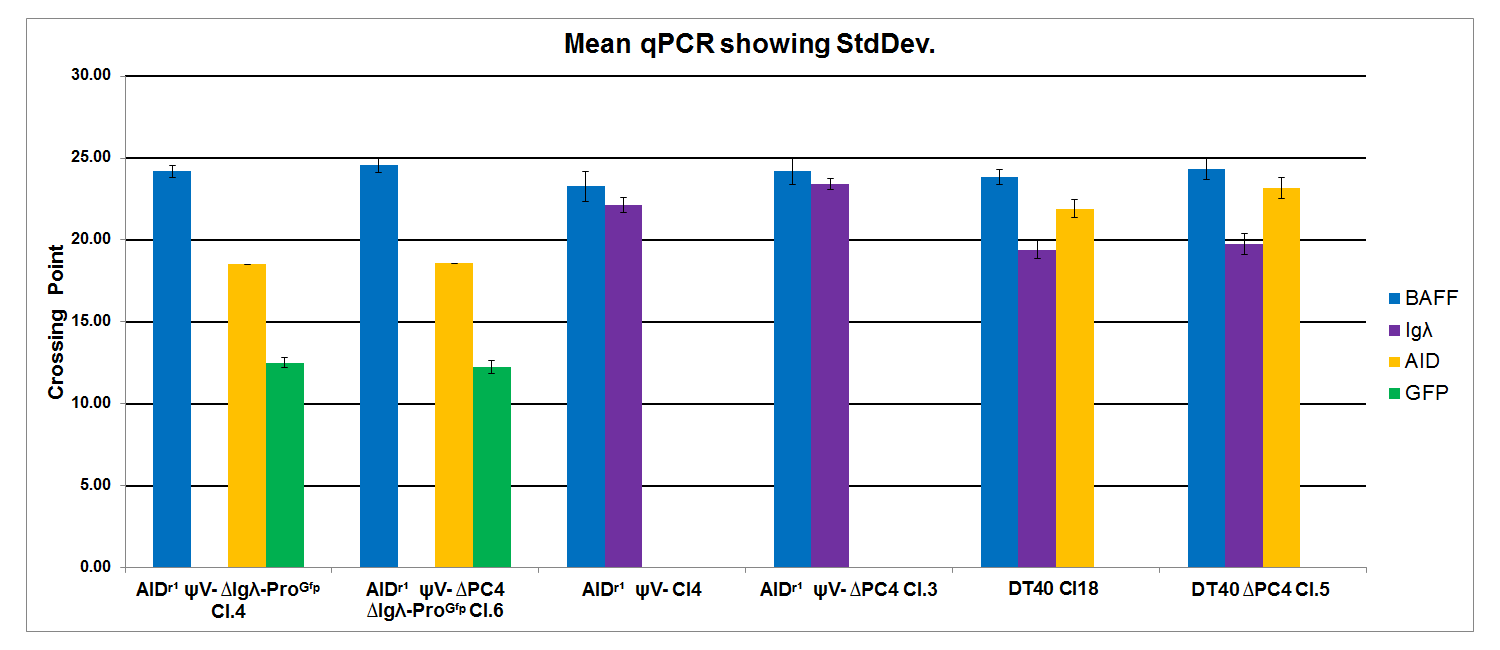
**

**Expression qPCR analysis to rule out transcriptional effects.**

Total mRNA was isolated concurrently with bulk gDNA isolation and tested for AID, Igλ and GFP expression levels. B-cell Activating Factor (BAFF) expression was used for control. Mean qPCR crossing points are shown with standard deviation bars.

**Supplementary Fig 4**

**
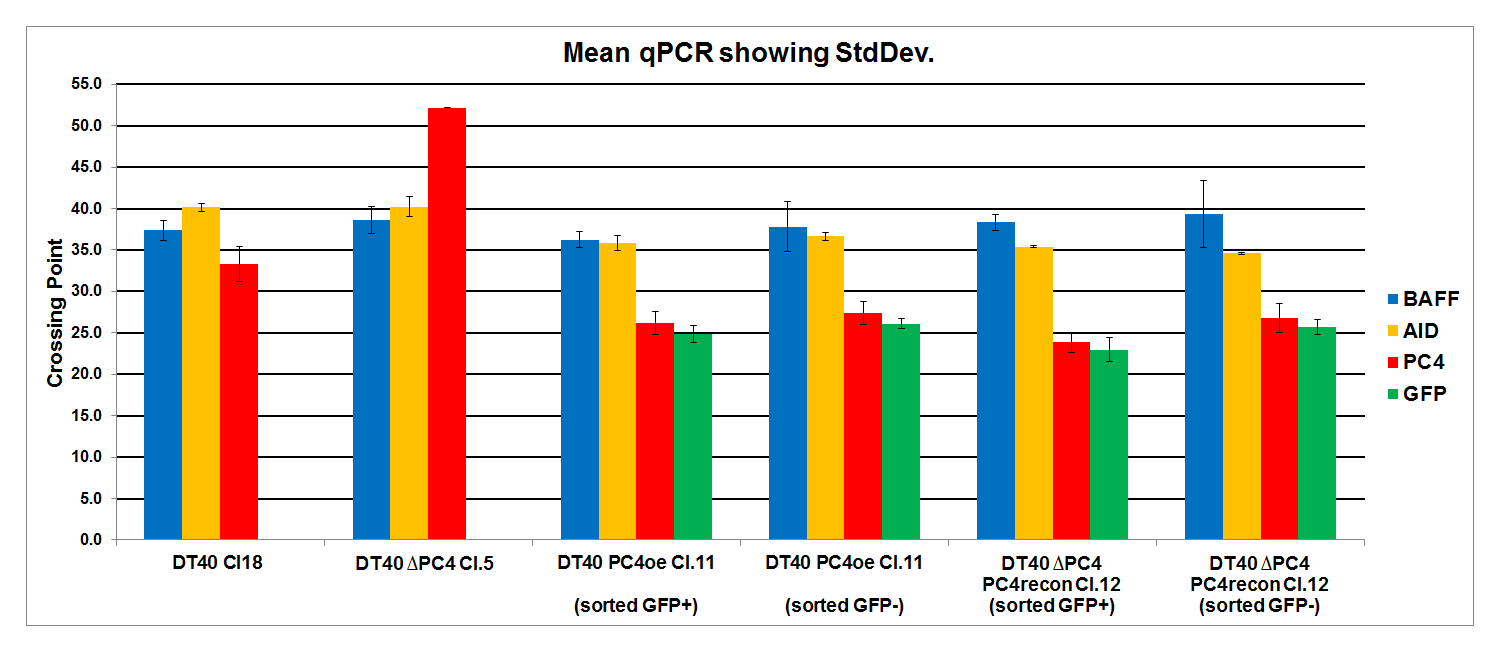
**

**Expression qPCR analysis to address PC4iresGFP transcript stability.**

Total mRNA was isolated from bulk & sorted populations and tested for PC4 and GFP expression levels. AID and B-cell Activating Factor (BAFF) expression was used for control. Mean qPCR crossing points are shown with standard deviation bars.

8 of 9 replicates failed to detect PC4 mRNA and 1 of 9 returned a value of 52.2.

**Supplementary Fig 5**


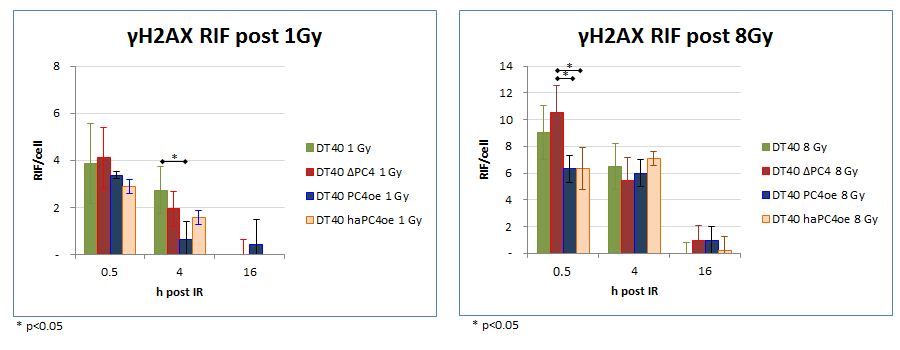


**Radiation-Induced γH2AX Foci (RIF) Analysis**

RIF analysis of WT DT40 Cl18 (DT40), DT40 ∆PC4, DT40 PC4oe and DT40 haPC4oe clones. Cells were sham irradiated (control values) and with 1 and 8 Gy. The values shown represent the mean of 3-4 independent experiments with control values subtracted (RIF). Error bars = standard deviation. Statistically significant (P< 0.05, *t*-test) differences are marked by bars.
